# Crimean-Congo Hemorrhagic Fever Virus: Current Advances and Future Prospects of Antiviral Strategies

**DOI:** 10.3390/v13071195

**Published:** 2021-06-22

**Authors:** Shiyu Dai, Fei Deng, Hualin Wang, Yunjia Ning

**Affiliations:** 1 State Key Laboratory of Virology and National Virus Resource Center, Wuhan Institute of Virology, Chinese Academy of Sciences, Wuhan 430071, China; daishiyu@wh.iov.cn; 2 Center for Biosafety Mega-Science, Chinese Academy of Sciences, Wuhan 430071, China

**Keywords:** Crimean-Congo hemorrhagic fever virus, bunyavirus, tick-borne virus, antiviral strategies

## Abstract

Crimean-Congo hemorrhagic fever virus (CCHFV) is a widespread, tick-borne pathogen that causes Crimean-Congo hemorrhagic fever (CCHF) with high morbidity and mortality. CCHFV is transmitted to humans through tick bites or direct contact with patients or infected animals with viremia. Currently, climate change and globalization have increased the transmission risk of this biosafety level (BSL)-4 virus. The treatment options of CCHFV infection remain limited and there is no FDA-approved vaccine or specific antivirals, which urges the identification of potential therapeutic targets and the design of CCHF therapies with greater effort. In this article, we discuss the current progress and some future directions in the development of antiviral strategies against CCHFV.

## 1. Introduction

Crimean-Congo hemorrhagic fever virus (CCHFV) is a tick-borne virus causing Crimean-Congo hemorrhagic fever (CCHF), a severe hemorrhagic fever in humans, with a mortality rate of up to 30% in more than 30 countries in Asia, Africa, southeastern Europe, and the Middle East [1]. The virus is transmitted to humans by the bites of infected ticks of the *Hyalomma* genus, or direct contact with tissue or body fluids from infected animals and humans [2]. A large variety of animals, such as cattle, donkeys, goats, hares, horses, ostriches, small rodents, and sheep, develop viremia without noticeable symptoms of illness following CCHFV infection [3], despite its high pathogenicity in humans. Following viral challenge, newborn mice, a subset of immunocompromised rodents and cynomolgus macaques could partially recapitulate the human disease and have been assessed as potential animal models of CCHFV [3,4,5]. The limits of appropriate animal models, as well as the requirement of such a biosafety level (BSL)-4 virus for high-containment laboratories, largely slow down the progress in virological study and development of antiviral drugs and vaccines.

CCHFV belongs to the genus *Orthonairovirus*, family *Nairoviridae*, order *Bunyavirales* [6]. The viral genome is composed of three negative-sense RNA segments. The small (S) segment encodes the nucleoprotein (NP), the medium (M) segment encodes the glycoprotein precursor (GPC) that is subsequently cleaved into mature Gn, Gc, and several nonstructural proteins including mucin, GP38 and NSm, and the large (L) segment encodes the L protein which contains the RNA-dependent RNA polymerase (RdRp) catalyzing viral RNA synthesis and an ovarian tumor (OTU) protease domain likely involved in viral antagonism of host innate immunity [7,8,9].

Due to the lack of specific antiviral therapies, high mortality rate, increased vector bionomics and climate change, CCHFV is considered an emerging arboviral zoonotic disease in many countries and is listed as a highly infectious pathogen that could cause a public health emergency. Thus, the development of novel antiviral therapeutics against CCHFV is urgently needed to manage the increasing public health threat of CCHF. Currently, supportive therapy is the primary mode of treatment. Ribavirin, a broad-spectrum antiviral medication, has been administered to human cases of CCHF; however, the therapeutic benefits remain elusive [10]. Other therapeutic candidates utilized in case reports include steroid, convalescent serum, and specific immunoglobulin, although there is no sufficient evidence to assess the efficiency of these medicaments [11]. In addition, potential inhibitors of bunyaviruses have been evaluated over the past decades and some of them have demonstrated possible efficacy to CCHFV infection. In this article, we review these current antiviral strategies of CCHFV (Table 1) and discuss some future directions for further research.

## 2. Current Antiviral Strategies

### 2.1. Nucleotide Analogues

Nucleoside analogues, a class of drugs targeting viral RNA polymerase, exhibit broad-spectrum antiviral activity against many distinct viruses both in vitro and in vivo. Many of them are currently being evaluated as candidate drugs for therapeutic use in emerging infections [35,36]. Several, including ribavirin, favipiravir and 2′-deoxy-2′-fluorocytidine, have also been used in anti-CCHFV tests.

#### 2.1.1. Ribavirin

Ribavirin is a purine nucleoside analogue compound with known broad-spectrum antiviral activity in vitro and in vivo, and is the only antiviral drug recommended by the World Health Organization (WHO) for the treatment of CCHF. In 1989, Watts et al. reported the anti-CCHFV effectiveness of ribavirin by in vitro tests with African green monkey kidney cell line (Vero cells), in which the drug significantly decreased the replication of various CCHFV strains [12]. The protective activity of ribavirin was then examined in CCHFV-infected suckling mice [13], signal transducer and activator of transcription-1 (STAT-1) knockout mice [14] and type I interferon receptor deficient (IFNAR^−^/^−^) mice [18,19], which suggested that ribavirin treatment against CCHFV must be started soon after infection to see clinical benefit. However, clinical evidence for the beneficial treatment of ribavirin is inconsistent and has attracted debate among researchers and clinicians [10,17]. Systematic reviews and meta-analyses showed insufficient efficacy of ribavirin for CCHF patients [15], or suggested that early treatment with ribavirin, <48 h after symptom onset, was needed for clinical benefit [16]. These various findings make the efficacy of ribavirin in CCHF patient inconclusive.

#### 2.1.2. Favipiravir

Favipiravir (also known as T-705) is licensed in Japan for the treatment of influenza virus infections but has shown promise against other highly pathogenic RNA viruses including severe acute respiratory syndrome coronavirus 2 (SARS-CoV2), Hantavirus, and Rift Valley fever virus (RVFV), as well as CCHFV [36,37,38]. Two groups have evaluated favipiravir against CCHFV in a type I interferon-deficient mouse model and both showed that favipiravir treatment effectively suppresses viral replication in several tissues and can reduce mortality following diverse CCHFV strain infections [18,19]. Work by Oestereich et al. has shown that favipiravir treatment is efficacious in inhibiting viral replication and preventing a lethal outcome following CCHFV infection, even when treatment is started two days before the expected time of death [19]. Furthermore, they also demonstrated that favipiravir and ribavirin exert synergistic effects in vitro, allowing lower doses of both drugs to be used in vivo with clinical efficacy, suggesting that a combined treatment yields beneficial rather than adverse effects [19]. In the study by Hawman et al., favipiravir showed clinical benefit even when administered six days post-infection, a time point at which mice were exhibiting advanced disease, including death [18]. Their further study demonstrated that favipiravir has therapeutic efficacy against CCHFV infection in a cynomolgus macaque model. However, compared to the results from the mouse model, favipiravir treatment only showed a modest benefit in CCHFV-infected cynomolgus macaques, even though treatment was initiated 24 h post-infection, which may be attributed to the subcutaneous administration of favipiravir and nonuniform lethality of the cynomolgus macaque model [20] and needs further investigation to be clarified. Nevertheless, overall, these data suggest that favipiravir may be an effective antiviral for the treatment of advanced CCHF.

#### 2.1.3. 2′-deoxy-2′-fluorocytidine

In addition to ribavirin and favipiravir mentioned above, several other prominent broad-spectrum inhibitors against bunyavirus infections also have been reported. Welch et al. developed a high-throughput screening assay for CCHFV antiviral drug discovery using a recombinant CCHFV encoding a reporter protein. They identified a compound, 2′-deoxy-2′-fluorocytidine (also known as 2′-fluoro-2′-deoxycytidin, 2′-dFC) as a potential CCHFV antiviral, with inhibitory activity superior to that of favipiravir or ribavirin in vitro. They also demonstrated that 2′-dFC acts synergistically with favipiravir to inhibit CCHFV replication without causing cytotoxicity, suggesting the potential of a combination therapy with 2′-dFC and favipiravir [21]. Smee et al. further investigated the potential of 2′-FdC as a broad-spectrum inhibitor of bunyaviruses in the IFNAR^−^/^−^ mouse model [39]. They indicated that 2′-FdC has inhibitory effectiveness against both RVFV and severe fever with thrombocytopenia syndrome virus (SFTSV) and is thus a promising candidate for treating certain bunyavirus infections [39]. However, how 2′-FdC performs in animal models of CCHFV infection remains unclear.

### 2.2. OTU Protease Inhibitors

Many viruses encode deubiquitinating (DUB) enzymes that play an important role in viral replication and innate immune evasion. The CCHFV L protein contains an OTU domain also exhibiting DUB activity, which binds and removes ubiquitin (Ub) and interferon stimulated gene 15 (ISG15) from their protein targets to circumvent host antiviral innate immune responses [7,40]. This makes the DUB active site of CCHFV OTU a highly attractive drug target, as disrupting the activity is expected to not only directly interfere with viral replication but also enhance immune responses upon infection. Kocabas and Aslan developed a fluorescent reporter assay of CCHFV OTU protease to screen CCHFV OTU inhibitors that might possess potential antiviral activity against CCHFV. They showed noticeable OTU inhibitory activities of two molecules (especially the compound phenanthrenequinone) in vitro [22]. Zhang et al. screened a phage display library of billions of Ub variants (UbVs) and showed that a UbV-CC4 could bind the CCHFV OTU with high levels of affinity and specificity in vitro and selectively blocked the deubiquitinating and deISGylating activities of CCHFV DUB [23]. Scholte et al. further demonstrated that stable occupancy of CCHFV OTU by UbV-CC4 strongly inhibits viral growth not only by inhibiting the IFN antagonist function of OTU but also by blocking the formation of RNP complexes [24]. These studies indicate the dual antiviral potential of targeting CCHFV OTU and provide an important conceptual basis for the development of OTU-specific antiviral drugs. Since the data of the antiviral potential of CCHFV OTU inhibitors were generated in vitro, further in vivo studies should be necessary.

### 2.3. Interferon (IFN)

The innate immune response is the first line of host defense against viral infections in mammalian cells [41,42]. Previous studies have shown that CCHFV infection results in the substantial secretion of type I interferons (IFNs), especially IFN-α and IFN-β, and subsequent upregulation of interferon-stimulated genes (ISGs) [43,44]. In the studies by Andersson et al., pre-treatment with IFN-α was shown to have antiviral activity against CCHFV in vitro, most likely due to the induction of ISGs such as MxA [45,46]. However, established CCHFV infection is almost insensitive to subsequent treatment with IFN-α and moreover, no positive clinical data is available on IFN use against CCHF by far [26,46]. In the only clinical application case, patients and contacts were administrated with human IFN-α at 1985 in South Africa, but it was discontinued due to the serious side-effects [25]. Recently, Bordi et al. demonstrated that type III IFN (IFN-λ1) also has an anti-CCHFV activity, although it seems to be less effective compared with type I IFN (IFN-α). In addition, an obvious antagonistic effect between IFN-λ and IFN-α was observed in human A549 and Huh7 cell lines and remains to be elucidated [27].

### 2.4. Antibody Therapy

With antibody engineering advancing, antibody therapy has been growing steadily in recent years. Antibodies, especially monoclonal antibodies (mAbs), have been demonstrated to be effective in the treatment of several hemorrhagic fever virus-related infectious diseases with in vitro and in vivo models, including those caused by Ebola and Lassa viruses [47,48].

#### 2.4.1. Convalescent Serum

In the case of CCHFV, immunotherapy has demonstrated some clinical benefits in small studies. Vaccine studies in mice have shown that antibodies play an important role in preventing CCHFV infection [49,50]. Convalescent serum from donors has been used in several cases of CCHF patients and especially administering by the intravenous infusion, at earlier time, for longer duration, and in volumes greater than 200 mL could result in a better outcome [11]. Specific human immunoglobulin (CCHF-Venin), derived from the plasma pool of boosted donors, was tested in seven CCHFV patients in Bulgaria and showed good therapeutic effects [51]. However, the effectiveness of convalescent serum has not been evaluated in randomized controlled clinical trials with large sample sizes and no studies have proven the efficacy of specific immunoglobulin for the post-exposure prophylaxis or treatment of CCHF [30]. Moreover, there are still a number of challenges that prevent the large-scale adoption of convalescent serum, such as limited sources, individual differences and the complexity of blood products. Therefore, serum or immunoglobulin from convalescent patients is usually used in emergencies.

#### 2.4.2. Monoclonal Antibodies

In comparison with the convalescent serum from donors, neutralizing mAbs have high specificity, low immunogenicity and limited metabolic side effects and represent a promising therapeutic approach for the development of an effective treatment. Bertolotti-Ciarlet et al. generated a panel of murine mAbs specific to the glycoprotein pre-Gn or Gc. They found that mAbs to Gc, but not Gn, neutralize CCHFV in SW-13 cells. However, only a subset of Gc mAbs exhibited a protective effect in mice after passive immunization, whereas some non-neutralizing Gn mAbs protected suckling mice from a lethal challenge with the CCHFV strain IbAr10200, suggesting that antibody activities against CCHFV depend not only on the neutralizing properties, but also on host factors and non-neutralizing antibody-dependent mechanisms [29]. The same group also characterized the broadly cross-reactive neutralizing activity of their previously described murine mAb 11E7 [28]. Zivcec et al. further confirmed three anti-Gc murine mAbs (8A1, 11E7, and 30F7) showing cross-neutralization potency against CCHFV in vitro using a virus-like particle system in biosafety level 2 conditions and identified peptide binding regions of them [30]. Golden et al. then investigated the protection of these mAbs reported to target CCHFV pre-Gn or Gc using adult type I IFN-deficient mice. They demonstrated that, interestingly, a non-neutralizing antibody actually binding to GP38 (a secreted nonstructural glycoprotein in CCHFV), 13G8, protects the mouse model against lethal CCHFV infection [31]. Considering the immune rejection of human immune system to animal-derived antibodies, these murine mAbs need further humanized modification if they are used in clinic. In comparison, mAbs from patients are safer and worth isolation and functional testing. Furthermore, the use of a single antibody often only has limited efficacy and may be easy to induce tolerance. Strategies, e.g., antibody cocktail therapy and antibody–drug coupling, which can improve the biological activity of antibodies, will enhance the efficacy of antibody therapy. During the revision of this review, Fels et al. reported the isolation of a panel of neutralizing mAbs targeting CCHFV glycoproteins from convalescent donors [32]. To generate antibodies with increased antiviral activity and limited risk to induce viral escape mutation, they engineered bispecific antibodies bearing variable domains from two antibodies with a synergistic effect and successfully identified one antibody, DVD-121-801, that affords both prophylactic and therapeutic potential against CCHFV challenge in IFNAR^−^/^−^ mice [32].

Although antibody treatment for CCHF remains in its infancy, this approach may be an effective therapy against CCHFV in the absence of approved drugs.

### 2.5. Other Potential Antivirals

Targeting host cell pathways supporting viral replication is an attractive approach for the development of antiviral intervention strategies. CCHFV enters the cells via a clathrin- and early endosome-dependent pathway, which can be potential targets of treatments for infections [52]. Ferraris et al. investigated the anti-CCHFV activity of FDA-approved molecules targeting endocytic pathways. In their in vitro antiviral testing, chloroquine and chlorpromazine that interfere with the clathrin/pH-dependent endocytic pathway were identified as potential antiviral drugs for CCHFV [33]. Chloroquine and chlorpromazine are widely used anti-malarial or anti-psychotic drugs, respectively. The two compounds also show antiviral activity against multiple viruses, including members of the flaviviruses, retroviruses, and coronaviruses [53,54]. Chloroquine and chlorpromazine both blocked a post-entry step in CCHFV replication cycle on two permissive cells—Vero E6 and Huh7. Chloroquine was found to be the more selective CCHFV inhibitor with a selective index of more than 20. However, the low selectivity index of chlorpromazine diminishes its potential use in clinic, particularly if this drug is used alone. Chloroquine or chlorpromazine and ribavirin combination assays demonstrated synergistic effects, suggesting that combinatorial treatment may be a better strategy to control CCHFV infection. The efficacy of these two FDA-approved drugs against CCHFV infection should be further confirmed in animal models.

Tampere et al. established an image-based phenotypic high-throughput screening assay coupled with automated image analysis and tested a set of in-house small molecule inhibitors targeting oxidative stress and nucleotide metabolism pathways. The newly identified antiviral molecules, TH3289 and TH6744, exhibited broad antiviral activity against emerging RNA viruses including SARS-CoV-2, Ebola virus (EBOV), Hazara virus, as well as CCHFV, likely due to its multifaceted effects on cellular heat shock protein 70 (HSP70) pathways [34].

RNA interference using small interfering RNAs (siRNAs) to silence genes might be used to regulate viral replication by targeting cellular processes such as those aforementioned or directly by targeting viral genes. Foldes et al. designed chemically synthesized siRNAs targeting viral genes that could inhibit CCHFV replication in vitro [55]. Therein, they identified effective siRNAs targeting all the three segments of the CCHFV genome, providing support for the potential use of RNA interference techniques in the rational design of anti-CCHFV drugs.

## 3. Conclusions and Prospects

CCHF is a medically important tick-borne viral disease of humans with wide prevalence and is listed by the WHO as one of the top priority diseases for research and development in public health emergency contexts (https://www.who.int/activities/prioritizing-diseases-for-research-and-development-in-emergency-contexts, accessed on 21 June 2021). At present, medical countermeasures against CCHF remain controversial or experimental and the efficacy and safety of potential anti-CCHFV drugs also need comprehensive evaluation in standardized clinical trials. As a BSL-4 pathogen, CCHFV is strictly restricted to the special containment facilities for experimental manipulations of infections; moreover, suitable animal models also need to be further developed and optimized. Currently, they hamper virological studies and the assessments of prophylactic and therapeutic measures.

Since screening for antivirals in high-containment biosafety level settings is challenging, researchers have developed alternative methods. The entry-competent virus-like particle (tecVLP) system and recombinant fluorescent reporter virus which can be performed in the BSL-2 laboratory have been used in the initial screening of antivirals against CCHFV [21]. The identification of small molecule compounds inhibiting viral RNA synthesis can be conducted firstly with in vitro screening systems that rely on the availability of recombinant L protein or OTU protease and thus do not require high biosafety measures either [9,22]. In addition, computational virtual screening procedures provide both an alternative and a supplement to tiresome high-throughput screening, giving researchers the opportunity to hasten, facilitate and innovate the effectiveness of the overall drug discovery process. In the study by Sharifi et al., screening of FDA-approved drugs in silico indicated that doxycycline and minocycline are putative inhibitors of CCHFV NP [56]. Mirza et al. established an extensively refined homology model of CCHFV RdRp, which allowed the in silico predication of potential antiviral compounds [57]. The resolved structures of several critical CCHFV proteins, including NP, the OTU domain of L, and glycoprotein (GP38), have and will continue to facilitate viral protein-targeting drug discovery based on structure and computational approaches [58,59,60,61].

In order to advance anti-CCHFV therapy development, animal models of CCHFV infection that can exhibit clinical signs similar to human disease are necessary in pre-clinical studies. Two adult mouse models of lethal CCHFV infection which lack the cell-surface IFN-I receptor (IFNAR^−^/^−^) or the transcription factor essential for IFN signaling, STAT-1, have been reported and used for CCHFV pathogenesis and drug evaluation [3]. A lethal hamster model with a signal transducer and activator of transcription-2 (STAT-2) knockout has also been recently established. The efficacy of ribavirin against CCHF mortality shown in this hamster model demonstrates the model’s ability to be used for the evaluation of promising CCHFV therapeutic candidates [5]. Apart from the immunocompromised rodents, a lethal humanized mouse model transplanted with human hematopoietic CD34+ stem cells [62] and an immunocompetent mouse model which developed disease following infection with a mouse-adapted variant of CCHFV have been reported [63]. Moreover, an immunocompetent cynomolgus macaque model has also been developed and used for the evaluation of the efficacy of favipiravir treatment against CCHFV infection [4,20]. This non-human primate model merits further tests and optimization in the evaluation of countermeasures against CCHFV, e.g., in experimental settings with more appropriate administration approaches and viral dosages. Better understanding of virology and virus–host interactions in the future may provide new clues for development of engineered animal models with specific virus-infection-associated host factor humanized, which are supposed to have significant advantages compared to those with common immune signaling proteins simply deleted. While current anti-CCHFV therapies have been assessed with limited animal models and clinical trials, future directions should focus on developing more appropriate animal models of CCHFV for the pre-clinical study of therapeutics.

Given the time-consuming nature of antiviral drug development and approval, repurposing the use of existing drugs in other conditions could be a strategy. For most of these drugs, ample experience is available with dosing in humans. Moreover, their safety, absorption, distribution, metabolism, and excretion profiles are well-known. The clinical drugs for non-viral disease, chloroquine and chlorpromazine, have been shown to be efficient in inhibiting CCHFV in vitro [33]. Despite the diversity of viruses, there are several common stages in the viral life cycle, including entry, biosynthesis, assembly, and release. The similar host processes and proteins exploited by virus usually serve as promising targets for the design of broad-spectrum, host-directed antiviral drugs. A notable challenge for the development of antiviral drugs is the virus mutation, especially for CCHFV, which is an RNA virus with a high mutation rate. Selective pressure from therapeutics targeting viral components could increase the probability of escape mutation, thus leading to the emergence of drug-resistant strains, while intervention strategies directing host factors would be more rigid to selective escape pressure and might have broad-spectrum antiviral potential. However, it should be noted that host proteins themselves usually have crucial cellular physiological functions; therefore, more comprehensive evaluation needs to be considered when targeting them for antiviral intervention. On the other hand, protein sequence and structure are similar among viruses from the same genus. Some vital viral proteins involved in viral infection, such as the catalytic domain of RNA polymerases, can also be considered as promising targets for the development of pan-virus or pan-genus antivirals. In the case of CCHFV, the antivirals tested are mostly targeting RdRp and OTU protease, which themselves are notable targets for drug design; the discovery and development of inhibitors to other essential and conserved viral components in the CCHFV life cycle also need to be considered.

In view of many obstacles to progress, CCHF will clearly remain a significant public health threat for the foreseeable future. Further advances in areas such as structure analysis of viral proteins, immunocompetent animal model development, and the study of virus–host interaction would pave the way for effective medical countermeasures against CCHF. Additionally, successful research into CCHF therapeutics should also rely on collaboration among endemic countries.

## Figures and Tables

**Table 1:** Summary of antivirals against CCHFV.

Antiviral Name	Type	Target	In Vitro (Cell Line)	In Vivo	Effective Spectrum	Developmental Stage	Reference
Ribavirin	Small molecule	Viral RdRp	SW13, Huh7, Vero	Suckling mice; STAT-1 knockout mice; IFNAR^−^/^−^ mice; STAT-2 knockout hamster; cynomolgus macaque; certain clinical cases	RSV, IAVs, LASV, etc.	Clinical drug for HCV	[10,12,13,14,15,16,17,18]
Favipiravir	Small molecule	Viral RdRp	SW13, Huh7	IFNAR^−^/^−^ mice; cynomolgus macaque	RVFV, LASV, EBOV, ZIKV, SARS-CoV2, etc.	Clinical drug for influenza virus	[18,19,20]
2′-deoxy-2′-fluorocytidine	Small molecule	Viral RdRp	SW13, Huh7	--	RVFV, SFTSV, HCV, HSV, LASV, etc.	Clinical trial for HCV	[21]
Phenanthrenequinone	Small molecule	Viral OTU	--	--	--	Preclinical	[22]
UbV-CC4	Protein	Viral OTU	A549	--	--	Preclinical	[23,24]
IFN-α	Protein	Immune system	A549, Huh7, HUVEC	certain clinical cases	HCV, IAVs, HSV, etc.	Clinical trial for HCV	[25,26,27]
IFN-λ1	Protein	Immune system	A549, Huh7	--	IAVs, ZIKV, RSV, etc.	Preclinical	[27]
8A1, 11E7, 30F7	Antibody	Viral Gc	SW13	Suckling mice	--	Preclinical	[28,29,30]
6B12, 11F6, 7F5, 8F10	Antibody	Viral Gn	SW13	Suckling mice	--	Preclinical	[29]
13G8	Antibody	Viral GP38	SW13	IFNAR^−^/^−^ mice	--	Preclinical	[31]
DVD-121-801	Antibody	Viral Gc	Vero E6	IFNAR^−^/^−^ mice	--	Preclinical	[32]
Chloroquine	Small molecule	Endocytosis and other pathways	Vero E6, Huh7	--	Flaviviruses, retroviruses, CoVs, etc.	Clinical drug for nonviral disease	[33]
Chlorpromazine	Small molecule	Endocytosis and other pathways	Vero E6, Huh7	--	EBOV, CoVs, etc.	Clinical drug for nonviral disease	[33]
TH3289, TH6744	Small molecule	Protein folding machinery	Vero	--	SARS-CoV2, EBOV	Preclinical	[34]

Abbreviations: RSV, respiratory syncytial adenovirus; IAV, influenza A virus; RVFV, Rift Valley fever virus; LASV, Lassa fever virus; EBOV, Ebola virus; ZIKV, Zika virus; SFTSV, Severe fever with thrombocytopenia syndrome virus; HCV, hepatitis C virus; HSV, herpes virus; CoV, coronaviruses; SARS-CoV2, severe acute respiratory syndrome coronavirus 2; SW13, human adrenal gland cell line; Huh7, human hepatocarcinoma cell line; HUVEC, human umbilical vein endothelial cell line; Vero, African green monkey kidney cell line; A549, human lung carcinoma cell line; Vero E6, African green monkey kidney cell line, clone E6; STAT-1, signal transducer and activator of transcription-1; STAT-2, signal transducer and activator of transcription-2; IFNAR^−^/^−^, interferon receptor knockout. --, no report or unclear.

## Data Availability

The data that support the findings of this study are available from the corresponding author upon reasonable request.
